# Dispersion Characteristics, the Mechanical, Thermal Stability, and Durability Properties of Epoxy Nanocomposites Reinforced with Carbon Nanotubes, Graphene, or Graphene Oxide

**DOI:** 10.3390/polym16131836

**Published:** 2024-06-27

**Authors:** Miraidin Mirzapour, Patrice Cousin, Mathieu Robert, Brahim Benmokrane

**Affiliations:** Department of Civil and Building Engineering, University of Sherbrooke, Quebec, QC J1K 2R1, Canada; miraidin.mirzapour@usherbrooke.ca (M.M.); patrice.cousin@usherbrooke.ca (P.C.); brahim.benmokrane@usherbrooke.ca (B.B.)

**Keywords:** polymer matrix, mechanical properties, microstructural analysis, plastic deformation

## Abstract

Carbon-based nanoparticles (CBNs) are regarded as promising nanofillers in nanocomposites to produce high-performance fiber-reinforced polymers (FRPs). To date, no systematic investigations have been carried out on the structural variations of nanofillers and their influences on dispersion characteristics, which give nanocomposites their mechanical and durability properties. Moreover, environmentally unfriendly organic solvents are used to exfoliate and disperse CBNs in a polymer matrix. This study developed a green, easy approach to preparing epoxy/CBN nanocomposites. We demonstrated graphene oxide’s (GO) effective dispersion capacity, creating good interface interaction that dramatically influenced properties at loadings as low as 0.4 wt%. The tensile strength and toughness of the epoxy increased by about 49%; and 160%, respectively. Incorporating 0.4 wt% of multi-wall carbon nanotubes (MWCNTs), graphene nanoplates (GNPs), or GO into the epoxy increased the modulus storage by around 17%, 25%, and 31%, respectively. Fractography analysis of fracture surfaces indicated the primary reinforcing mechanisms (crack deflection and penning) as well as the secondary mechanism (bridging effect) enhancing the mechanical characteristics of nanocomposites. Incorporating GNPs, GO, or MWCNTs into the epoxy decreased the water absorption at saturation by about 26%, 22%, and 16%, respectively.

## 1. Introduction

Polymer nanocomposites are a class of nanomaterials in which one or more nanostructured materials are incorporated in the polymer matrix to achieve a new material with greater properties than a regular composite. These nanocomposites are applied in a wide variety of technologies and different industries, such as aerospace, automobiles, construction, film packaging, electronics, sensors, and batteries [1,2,3]. Advancements and continual research in nanotechnology promise the development of more potential nanocomposites with varied useful applications. Although a significant amount of research is being conducted on polymer nanocomposites, manufacturing an appropriate nanocomposite with high quality and performance, introducing acceptable exfoliation of nanoparticles, and creating linkages between the nanoparticles and the polymer matrix remain challenging [4].

Fiber-reinforced polymer (FRP) composites have recently gained popularity for a wide range of engineering applications, particularly civil engineering. FRP reinforcing bars are perfectly suited for structural applications in concrete structures and are an adequate substitute for steel bars thanks to their exceptional mechanical properties, high durability, and high strength-to-weight ratio [5,6,7,8,9]. FRP composites have been applied successfully to repair and rehabilitate reinforced concrete structures such as columns since 1990. Afterwards, this application extended to concrete bridge decks, beam-column joints, shear walls, tanks, and pipes [10,11]. FRP composites are composed of fibers impregnated with a polymer matrix to generate a strong, durable structure. The fibers supply high fatigue resistance, stiffness, and tensile strength, while the polymer matrix protects against environmental factors such as ultraviolet radiation and moisture [12,13]. One of the best ways to achieve high-performance composites is to improve the mechanical and thermal properties, as well as the durability of polymer hosts [14,15,16,17,18]. Epoxy (EP) resins are thermosetting resins that have been widely used in coatings, adhesion, construction structures, and electronics due to their outstanding bonding and chemical resistance. Cured epoxy resins have a high crosslink density, which leads to high modulus and strength. Still, they are very brittle, resulting in undesirable toughness and unfavorable resistance to crack creation and propagation. Moreover, FRP composites are usually used in harsh environments, hence, the resistance of epoxy to corrosion and environmental degradation should be improved. The existing epoxy resin can barely meet the particular performance requirements of increasing demands. Nanofillers have recently been studied to improve the mechanical and thermal properties as well as the durability of thermosetting resins [19,20,21,22,23,24,25,26]. Mirzapour et al. have used nanosilica and CNT to improve the mechanical and thermal stability of FRP composites [12,22]. The SEM pictures showed that nanosilica particles during the flame retardancy test, by increasing the temperature, milted and covered the fiber and acted like a secondary thermal protection system [12]. On the other hand, the CNTs produced a network char layer when the pyrolysis reaction started. It covered the produced char residue and acted as an anti-oxidizing agent and a thermal protective barrier, forming a carbon-based network [22]. In a study on graphene epoxy composites, a noticeable increment of 31% in modulus and 40% in fracture toughness was observed [27]. Yavari et al. have reported a dramatic increase in the fatigue life of hierarchical graphene composites [28]. Amer et al. have used graphene to improve the long-term durability of hybrid flax fiber-reinforced polymers and investigated its effect on the mechanical performance of composites [29].

Carbon-based nanoparticles (CBNs)—especially one-dimensional (1D) carbon nanotubes (CNTs) and two-dimensional (2D) graphene sheets—deliver outstanding mechanical, thermal, and electrical properties [20,30]. CNTs have a high aspect ratio, sp^2^ hybridized filler, while graphene is a 2D single-atom-thick layer of carbon [31,32]. To derive the exceptional mechanical and functional properties of carbon-based nanofillers, nanoparticles should be completely exfoliated and homogeneously dispersed in a polymer matrix [33]. Nanofiller clusters or agglomerations result in insufficient interface area with undesirable interfacial strength. Consequently, they often compromise their reinforcing or toughening influence. 

Various methodologies are being applied in studies to avoid restacking or reaggregating nanoparticles, enhancing their dispersion in polymer matrices. Some examples are the combination of mechanical mixing, microwaving, and chemical surface modification with or without organic solvents at elevated temperatures [34,35,36]. Organic solvents are commonly used in the processing of graphene as they can wet the graphene surface, migrate into the layer spacing, and create space for exfoliation [37,38]. Removing the residual solvents and surfactants is not easy and entails a complicated process, thereby considerably increasing expenses and seriously hindering the practical development and utilization of FRP bars. Moreover, the process occasionally requires the evaporation of toxic, environmentally unfriendly organic solvents [39]. Therefore, producing well-dispersed carbon-based nanofillers without organic solvents by using short-time sonication and eco-friendly methods would be preferable.

Other common methods for preparing polymer/CBN nanocomposites are associated with nanoparticle surface modification, but except for some special applications such as electronics and aerospace, they are not appropriate for the industrial production of FRP bars or the general application of composites, mainly because these methods are very expensive and environmentally unfriendly [40,41,42,43,44]. Another method of solving this problem that has been considerably investigated is the surface functionalization of graphene and graphene oxide [45,46]. Nevertheless, most of the methods for modifying the surface of GO are very complicated and expensive. Moreover, they still need to use organic solvents, which are not eco-friendly.

Recently, studies have been conducted to develop organic solvent-free and surfactant-free yet effective methods for polymer nanocomposites. Mirabedini et al. applied solvent-free processing techniques to directly exfoliate and disperse commercial GNPs in a polymer matrix [47]. Naeem et al. studied a thermal technique, followed by mechanical stirring, for exfoliating GNPs in liquid-state epoxy oligomers. Using this technique enabled them to considerably increase the thermal and mechanical properties of an epoxy resin [48]. In our previous study, we used a solution-mixing process by introducing a novel binary solvent system for well-exfoliated graphene/vinyl ester resin nanocomposites [49]. The results revealed that well-exfoliated graphene considerably increased the mechanical, thermomechanical, and durability properties of the prepared nanocomposites. In this research, we applied different carbon-based nanoparticles without using organic solvents for synthesizing epoxy nanocomposites.

In our study, multi-wall carbon nanotubes (MWCNTs), graphene nanoplates (GNPs), and as-synthesized graphene oxide (GO) synthesized from GNPs were used as the representative one- and two-dimensional nanofillers, respectively. Nanoparticles were dispersed in a curing agent of epoxy resin by high shear mixing and bath sonication procedures. X-ray photoelectron spectroscopy (XPS), X-ray diffraction (XRD), and scanning electron microscope (SEM) were employed to examine the structure and composition of the synthesized GO. The dispersion quality of nanofillers in epoxy resin was investigated using transmission electron microscopy (TEM) and scanning electron microscopy (SEM). The tensile test and resulting dynamic mechanical analysis (DMA) and water absorption were employed to evaluate the mechanical, thermomechanical, and durability characteristics of all the nanocomposites. Samples of pure epoxy were prepared for comparison.

## 2. Materials and Methods

### 2.1. Materials

Multi-walled CNTs (687804) and graphene nanoplates (GNPs) (900410) were selected as the CNTs and GNPs. Table 1 lists the key physical characteristics of the MWCNTs and GNPs. Epoxy (EP) resin EPX TFC (1000R) with a viscosity of 600–750 cps and a curing agent (polypropylene glycol (PPG)-based polyetheramine) with a viscosity of 90 cps from Resoltech (Marseille, France). KMnO_4_, H_2_SO_4_ (95%), H_3_PO_4_ (85%), H_2_O_2_ (30%), HCl (30%), and ethanol were purchased from Aldrich (St. Louis, MO, USA) and used as received.

### 2.2. GO Sheet Synthesis 

Graphene oxide powder was synthesized from GNPs using the Tour method. Compared to the Hummers method, the Tour method is safer, has no toxic gases, and provides easy temperature control [50]. 

### 2.3. Freeze-Dried Graphene Oxide

We used a novel approach to obtain a well-exfoliated GO nanosheet. The as-prepared graphene oxide was suspended in deionized water and sonicated (5 s on-pulse; 2 s off-pulse) at 70% amplitude, by an ultrasonic probe sonicator (Q-Sonica with 5 mm microtip, Newtown, CT, USA) for 2 h. After that, the graphene oxide aqueous dispersion was frozen for 24 h. Finally, the prepared graphene oxide was vacuum dried with a freeze-drying apparatus (VirTis SP Scientific Sentry, Warminster, PA, USA) for 48 h. The ice was removed by sublimation under vacuum, leaving a fluffy GO powder, leading to highly exfoliated GO that existed as a very thin layer (details given below).

### 2.4. Nanofiller Dispersion

We used a green method to disperse the nanofillers that involved no solvents or surfactants that were not environmentally friendly. Instead, the nanoparticles were dispersed in the hardener because their viscosity was much less than that of the epoxy matrix. The dispersion of nanoparticles was performed as follows: In a typical process, 200 mg of GNP sheets were added to a flask with the required amount of curing agent and stirred at 1000 rpm for 30 min at room temperature before 1 h of sonication in a bath sonicator (Digital ultrasonic cleaner model, 40 kHz, 150 W, VIXEN, Windsor, ON, Canada). The black mixture was degassed in a vacuum oven for 1 h at 40 °C to remove the air bubbles.

### 2.5. Fabrication of Epoxy Nanocomposites

Epoxy/CBN nanocomposites were fabricated with high shear mixing and bath sonication procedures. The nanoparticle/hardener mixture was added to the required epoxy resin (resin-to-hardener weight ratio of 100:50) and mixed for 1 h with a high-speed mixer at 7000 rpm to achieve the optimum doughnut effect. Then, the mixture was sonicated for 1 h in a bath sonicator. Subsequently, the mixture was degassed under vacuum at 30 °C for 1 h, poured into a mold, and cured at room temperature for 24 h. Table 2 summarizes the composition of the evaluated recipes.

### 2.6. Characterization

Water absorption. To measure the water absorption of the pure resin and nanocomposites, six samples of them were immersed in deionized water at 50 °C (ASTM D 570 [51]). Calculating the amount of absorbed moisture is carried out by Equation (1):

%M = 100 × (M_cond_ − M_dry_)/M_dry_ (1)

where %M is the mass gain due to moisture uptake, M_cond_ and M_dry_ are the masses after and before conditioning, respectively.

*X-ray diffraction (XRD).* XRD (Bragg-Brentano, X’Pert Pro PPD, Malvern PanalyticalLtd., Malvern, PA, USA) with Cu target radiation was used for characterizing the crystal structures of the graphene and GO. Wide-angle diffraction scanning was applied at 40 kV and 50 mA within the range of 5–50° and at a scanning rate of 3°/min.

*X-ray photoelectron spectra (XPS).* The XPS analyses were carried out with a Kratos Axis Ultra spectrometer (VG ESCLab 250, Manchester, UK) equipped with a hemispherical electron analyzer and a monochromatic Al Kα source (15 mA, 14 kV).

*Transmission electron microscopy (TEM).* TEM (HITACHI, model H-7500, Santa Clara, CA, USA) was used to examine the dispersity of nanoparticle in the nanocomposites. An ultramicrotome (LKB Nova, Bromma, Sweden) with a diamond knife was used to cut the nanocomposites. A thin section of nanocomposites with a 70 nm thickness was cut and then collected on 250 mesh copper grids for TEM observation.

*Scanning electronic microscopy (SEM*). SEM was used to evaluate the morphology and dispersion of the nanoparticles on the fracture surface of the nanocomposites. SEM micrographs were taken with an SEM microscope (Hitachi, model S-3000N) using the backscattering mode at 10 kV. All nanocomposites were polished and sputtered with gold.

*Tensile properties*. Tensile tests were measured using a Zwick/Roell (model Z050, Ulm, Germany) testing machine on dumbbell-shaped samples with a crosshead speed of 1 mm/s according to ASTM D-638 [52]. At least six specimens were tested for each composition, and the average values were calculated.

*Dynamic mechanical analysis (DMA).* Thermomechanical properties were analyzed using a DMA Q850 from TA Instruments (New Castle, DE, USA). All the samples were run at 1 Hz frequency, applying bending deformation with an amplitude of 15 μm, scanning from 30 to 200 °C, and a heating rate of 3 °C/min with the single cantilever bending mode. The maximum of tan δ versus temperature plots was used to identify the a-relaxation associated with the glass transition temperature (TG). Each measurement was repeated three times.

*Thermal stability.* The thermal stability of the specimens was evaluated by thermal gravimetric analysis under a nitrogen atmosphere with TA Instruments (model Q100 calorimeter). Three specimens were tested for each composition.

## 3. Results and Discussion

### 3.1. Characterization of As-Synthesized GO

**Figure 1:**
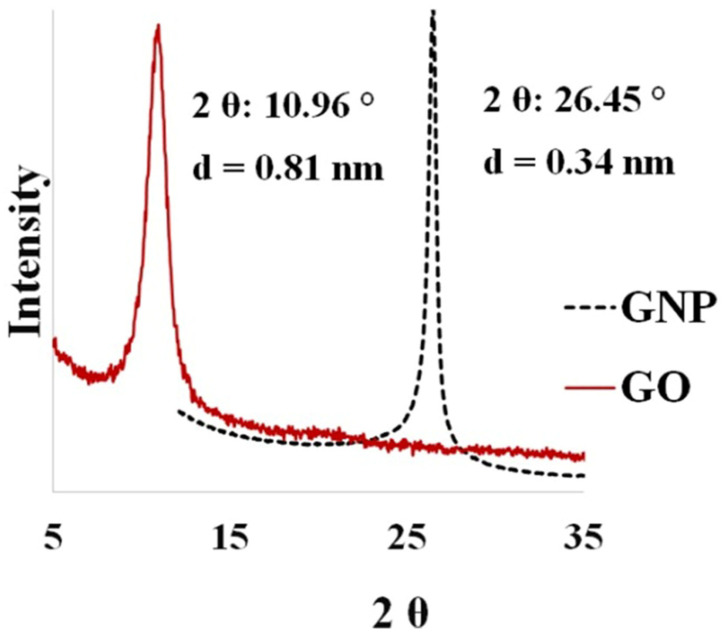
XRD of graphite and as-prepared GO.

*XRD*. Figure 1 shows the X-ray diffraction results of the GNP and as-synthesized GO. The XRD spectra of the GNP and synthesized GO yielded sharp diffraction peaks at 26.45° and 10.96°, respectively. The GNPs were composed of several stacked graphene nanosheets with an internal spacing of 0.34 nm. The Tour oxidation process generated carboxyl, epoxide, and hydroxyl groups on graphene oxide nanoparticles, and the d-spacing of the graphene oxide was enhanced to 0.81 nm [53,54].

**Figure 2:**
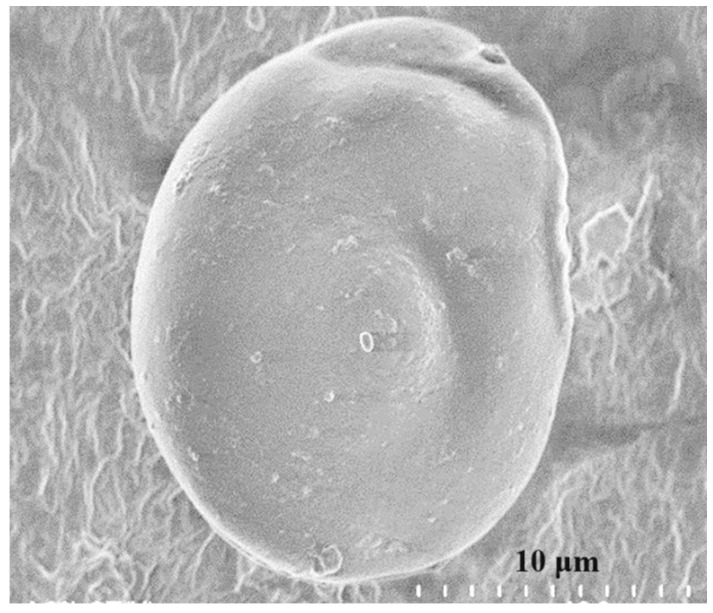
SEM micrographs of sonicated, as-prepared GO after 30 min of sonication.

*Microstructure of as-prepared GO*. SEM micrographs can provide more realistic knowledge about GO nanoparticles. It should be mentioned, however, that determining the precise size of graphene is very difficult due to the large surface area and strong attractive Van der Waals forces. Moreover, GO nanosheets are always susceptible to wrinkling, overlapping, and folding [53]. A novel method was used to obtain high-quality images of the prepared graphene oxide. First, 100 mg of GO, prepared by freeze-drying (Section 2.3), was dispersed in deionized water and exfoliated using the ultrasonic probe sonicator for 30 min. Then the suspension was poured on a thin film of alginate and air-dried at room temperature for 24 h. Figure 2 is an SEM micrograph of as-synthesized GO that was sonicated in water for 30 min, exhibiting relatively well-exfoliated GO nanoparticles and unfolded GO nanoparticles with a few fine wrinkles. It is an ellipse shape of GO with major and minor diameters of around 15 and 10 μm and a thickness around the nanometer, consisting of probably a few layers of graphene sheets.

**Figure 3:**
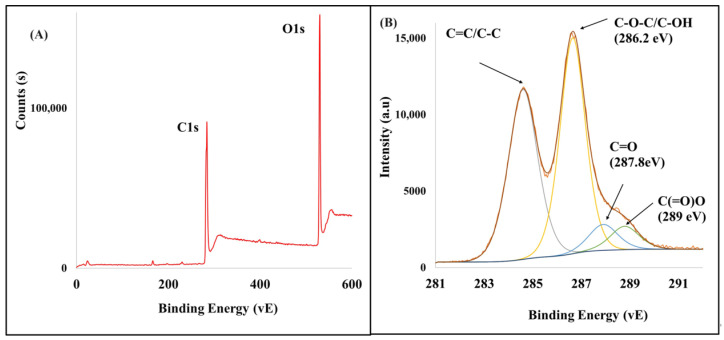
(**A**) XPS survey spectra of GO, (**B**) deconvoluted peaks of the C1s XPS spectrum.

*XPS.* The XPS spectra were investigated to further identify the surface chemical compositions of the as-prepared GO. Figure 3A shows that the GO had two peaks, including C1s (286 eV) and O1s (533 eV). As Figure 3B represents the C1s core level, the spectra of the GO were compared by deconvoluting each spectrum into four peaks corresponding to these functional groups: sp^2^ C=C (284.8 eV), C–O–C/C–OH (epoxy/hydroxyls) (286.2 eV), C=O (carbonyl) (287.8 eV), and O–C=O (carboxylates) (289.0 eV) [50]. The combination of all percentages of the oxidized materials shows that graphite oxide has almost 58% oxidized carbon and 42% graphitic carbon. 

### 3.2. Dispersion Validation

**Figure 4:**
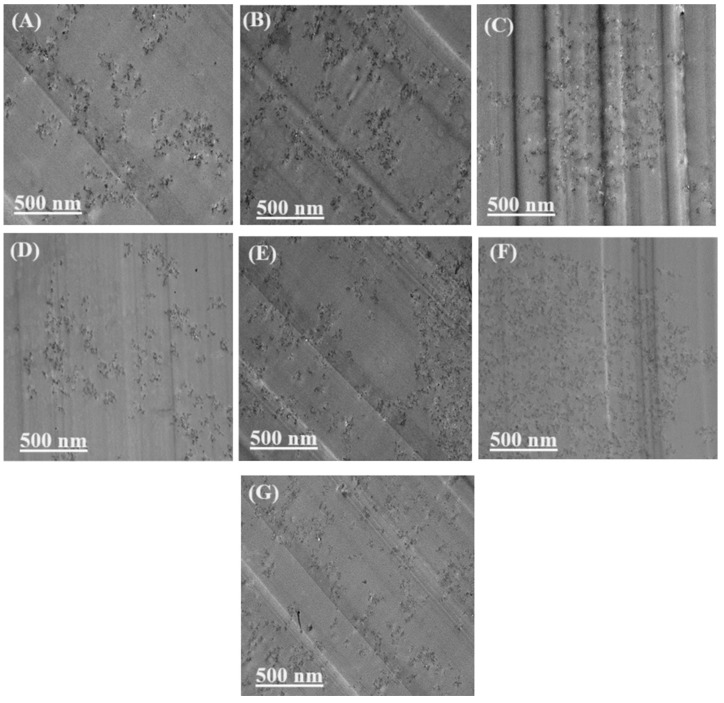
TEM micrographs of nanocomposites (**A**) EP/CNT 0.2, (**B**) EP/CNT 0.4, (**C**) EP/CNT 0.6, (**D**) EP/GNP 0.2, (**E**) EP/GNP 0.4, (**F**) EP/GNP 0.6, and (**G**) EP/GO 0.4.

TEM images can be used to verify the dispersion of nanoparticles in a polymer matrix. Because of structural differences, MWCNT nanoparticles tend to cluster and get entangled, while graphene and GO are prone to stacking and overlapping in multiple layers. Figure 4 shows the TEM micrographs of prepared nanocomposites. EP/CNT 0.2 (Figure 4A) and EP/GNP 0.2 (Figure 4D) show good dispersion without clear particle clusters or aggregations. Among EP/CNT 0.4 (Figure 4B), EP/GNP 0.4 (Figure 4E), and EP/GO 0.4 (Figure 4G), the latter has a comparatively homogeneous dispersion of GO nanoparticles with the smallest aggregation size. This high-quality dispersion of GO in the epoxy matrix can be explained by chemical interaction, hydrogen bonding, or even covalent bonding between the epoxy functional groups on GO nanoparticles and the amine groups of the curing agent [55,56,57,58]. Figure 4C clearly shows that the EP/CNT nanocomposite with 0.6 wt% MWCNTs does not display good dispersion. Nanoparticles are concentrated in the center of the image, with some particle clusters. In comparison, the epoxy/graphene nanocomposite with the same amount of nanoparticles (0.6 wt% of GNPs) had better particle dispersion without clear aggregation (Figure 4F). The good dispersion of MWCNTs, graphene, and GO in the polymer matrix—which we achieved in our research project without using organic solvents, surfactants, or the complicated surface modification of nanoparticles—has rarely been reported [59,60,61]. Zhang et al. achieved a comparatively uniform distribution of MWCNTs and graphene in epoxy resin with only small agglomerations of nanoparticles by grafting carboxymethyl cellulose on the surface of nanoparticles [62]. He et al. [63] and Guimont et al. [64] produced relatively acceptable GO distribution after surface modification of GO and dispersion in organic solvents. Xue et al. achieved acceptable dispersion of GO in epoxy resin by modifying the surface of GO. First, they directly mixed the aqueous GO dispersion with the TDE85/*N*,*N*-dimethylformamide (DMF) solution to prevent irreversible agglomeration [39]. Then, water and DMF were evaporated under heat. After that, the prepared surface of the modified GO was ground with a three-roll mill and mixed with epoxy resin. The high-quality dispersion of nanoparticles can lead to the creation of a greater interface with the polymer matrix for stress transfer, which considerably improves nanocomposite performance.

### 3.3. Tensile Properties

**Figure 5:**
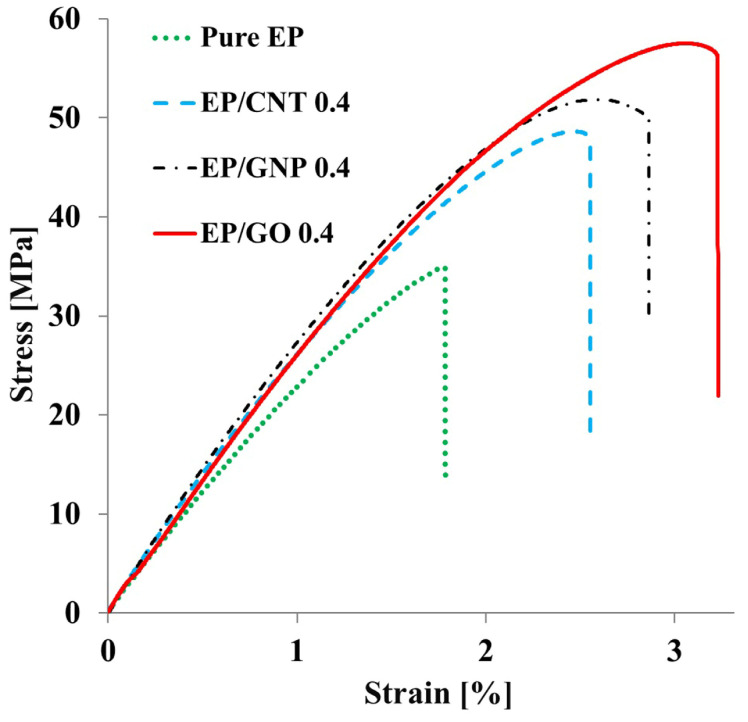
Representative stress-strain curves of pure EP and nanocomposites.

Figure 5 shows plots of the representative stress-strain curves of the pure epoxy and the nanocomposites EP/CNT 0.4, EP/GNP 0.4, and EP/GO 0.4. The pure epoxy exhibited a linear stress-strain relationship that finally led to an abrupt failure, which is a well-known characteristic of brittle materials. Although the nanocomposites—especially the EP/GNP and EP/GO—had a more or less elastic behavior, they reached a yield point, thereby explaining the plastic deformation before failure [65]. Some researchers have reported the plastic deformation of epoxy resin resulting from the incorporation of surface-modified graphene [66,67]. On the other hand, Zhang et al. studied the influence of surface-treated CNTs and graphene on the mechanical properties of epoxy resin and found that adding the surface-modified carbon-based particles improved the tensile strength of the matrix. Nevertheless, the as-prepared nanocomposite still exhibited brittle behavior like pure epoxy [45].

**Table 3:** Tensile properties of pure EP and nanocomposites.

Samples	Tensile Strength (MPa)	Young’s Modulus (GPa)	Elongation at Break (%)	Toughness ^a^ (MJ m^−3^)
Pure EP	37.2 (±3.1)	2.1 (±0.2)	1.81(±0.24)	0.66 ± 0.05
EP/CNT 0.2	41.5 (±1.9)	2.7 (±0.1)	2.65 (±0.13)	1.10 ± 0.03
EP/CNT 0.4	47.8 (±2.4)	2.8 (±0.1)	2.71 (±0.11)	1.30 ± 0.04
EP/CNT 0.6	46.2 (±2.6)	2.9 (±0.2)	2.67 (±0.31)	1.23 ± 0.05
EP/GNP 0.2	47.3 (±1.1)	2.5 (±0.1)	2.71 (±0.23)	1.28 ± 0.02
EP/GNP 0.4	49.2 (±2.3)	2.6 (±0.2)	2.83 (±0.17)	1.39 ± 0.04
EP/GNP 0.6	53.6 (±1.4)	2.7 (±0.1)	2.90 (±0.15)	1.55 ± 0.05
EP/GO 0.4	55.4 (±2.7)	2.6 (±0.1)	3.11 (±0.23)	1.72 ± 0.03

^a^ Calculated by integrating stress-strain curves.

Table 3 provides the results of tensile testing at room temperature, including ultimate tensile strength, Young’s modulus, strain at break, and toughness (area under stress-strain curve). The table shows that Young’s modulus, strength, and toughness of the pure epoxy are 2.1 GPa, 37 MPa, and 0.66 MJ m^−3^, respectively. Incorporation of the MWCNT nanoparticles considerably increased the tensile properties of the epoxy matrix. The highest tensile properties among EP/CNT nanocomposites belonged to the 0.4 wt% loading of CNT. The Young’s modulus, strength, and toughness of EP/CNT 0.4 are 2.7 GPa, 41.5 MPa, and 1.10 MJ m^−3^, respectively, corresponding to enhancements of 29%, 12%, and 67%, respectively, whereas these figures decreased as the content of MWCNTs increased to 0.6 wt% for EP/CNT 0.6. The enhancements occur due to the reinforcing effects of the well-dispersed carbon nanotubes, as observed in TEM pictures [22]. The drop in the tensile properties of EP/CNT 0.6 can be attributed to the fact that the MWCNTs probably acted as inclusions or defect centers rather than as reinforcement because of the weak dispersion of the nanoparticles and induced particle clusters (Figure 4C). Table 2 shows that the tensile properties of the EP/GNP nanoparticles—unlike the EP/CNT nanocomposites—increase by increasing the loading of graphene, reaching the maximum amount in EP/GNP nanocomposites. The Young’s modulus, tensile strength, and toughness of EP/GNP 0.6 are 2.6 GPa, 53.6 MPa, and 1.55 MJ m^−3^, respectively, corresponding to increases of 24%, 45%, and 135%, respectively. This constant improvement in the tensile properties of the graphene/epoxy nanocomposite can be related to the good dispersion and distribution of graphene sheets in the matrix (Figure 4D–F). Nevertheless, the epoxy/graphene oxide nanoparticles recorded our project’s maximum tensile properties among all as-received nanocomposites (Table 3 and Figure 5). The Young’s modulus, tensile strength, and toughness of EP/GO 0.4 are 2.5 GPa, 55.4 MPa, and 1.70 MJ m^−3^, respectively, corresponding to increases of 24%, 49%, and 160%, respectively. This can be attributed to the reinforcing effects of graphene oxide sheets and the improved interfacial interaction between the epoxy resin and GO nanoparticles. We achieved this considerable improvement in tensile characteristics by dispersing 0.4 wt% of pristine carbon-based nanoparticles into the curing agent. In contrast, the increase in tensile strength in the other epoxy nanocomposites containing more than 0.6 wt% of pristine and functionalized nanoparticles was relatively low (around 22%) [26,55]. The tensile-testing results show that dispersing carbon-based nanoparticles in the curing agent can be a promising approach to achieving high-performance nanocomposites with attractive mechanical properties. In our previous research, we tried to evaluate the effect of grafted GO or curing agent on the mechanical properties of epoxy resin by in-situ processing [68]. We predict the probable cure reactions in the GO/epoxy/hardener system that can increase the tensile properties of epoxy/GO nanocomposites. In that research, we synthesized GO from graphite powder with a diameter of around 30 μm, by sonicating the GO in water individual unfolded GO nanosheets were obtained. Increasing the sonication time to 60 min practically removed the folds; an individual very thin layer of GO, with some small overlaps, was obtained [68].

### 3.4. Fractography and Microstructure Analysis

**Figure 6:**
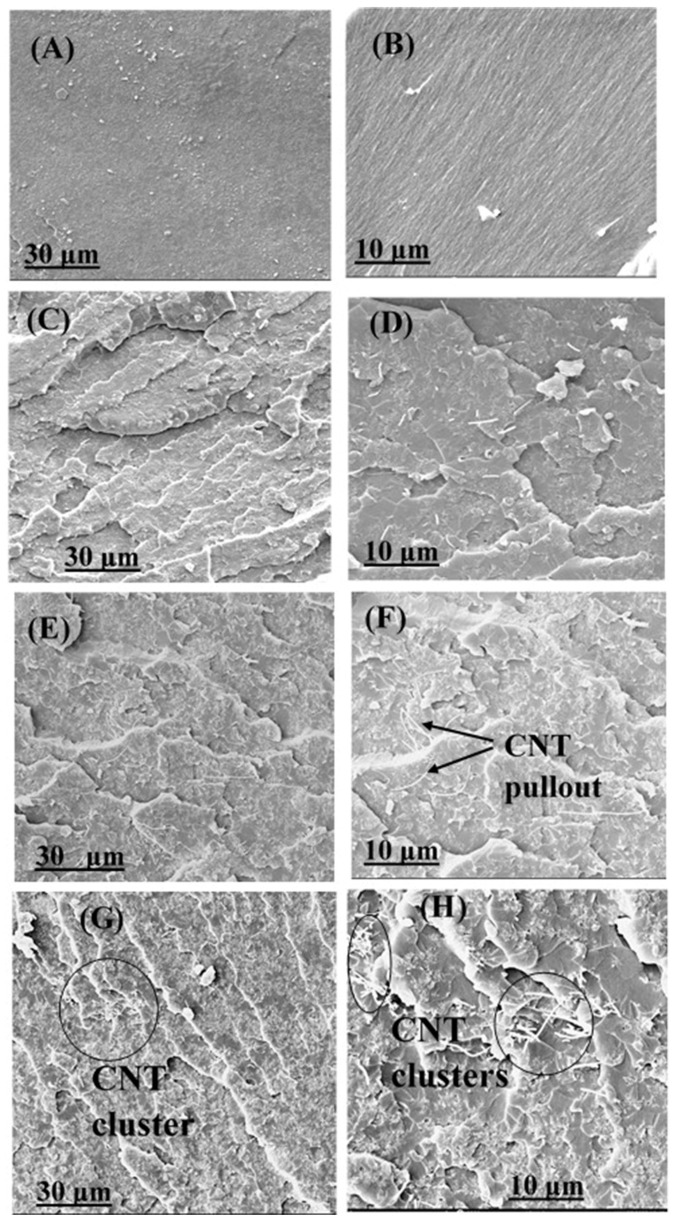
SEM micrographs of a fracture surface after tensile testing of (**A**,**B**) pure EP, (**C**,**D**) EP/CNT 0.2, (**E**,**F**) EP/CNT 0.4, and (**G**,**H**) EP/CNT 0.6.

After tensile testing, the epoxy composites were examined under SEM to further evaluate the influence of nanoparticle dispersion on the toughening mechanism. The fracture surface of neat epoxy displayed smooth surfaces, representing brittle fracture (Figure 6A,B). The incorporation of MWCNT nanoparticles remarkably changed the fracture surfaces of nanocomposites to coarse (Figure 6C–H). The generated cracks moved slowly and had to change direction to propagate. This phenomenon consumes more energy and causes a rougher fracture surface [26,69]. Therefore, the mechanical properties of nanocomposites increased compared to the neat polymer matrix. Moreover, Figure 6D illustrates a uniform dispersion of MWCNT nanoparticles in the epoxy matrix. This reveals that sonicating 0.2 wt% of MWCNT in the curing agent considerably broke up the MWCNT clusters into separated MWCNTs for good distribution in the matrix. While increasing the amount of MWCNT to 0.4 wt% still yielded good MWCNT dispersion without the formation of any clusters, it is obvious that some of the MWCNTs were pulled out of the surrounding epoxy matrix (Figure 6E,F). Some researchers found that the pulling-out phenomenon consumed a considerable amount of energy that further enhanced the toughness of the polymer matrix [70,71]. In contrast, increasing MWCNT incorporation up to 0.6 wt% led to poor dispersion of MWCNTs within the epoxy matrix (Figure 6G,H). There were some MWCNT aggregations, although not very dense, that could deteriorate the performance of nanocomposites. Nevertheless, we reached a high-quality dispersion of MWCNTs compared to other approaches [22,62]. For instance, Zhang and Huang found that surface modification of MWCNT reduced the cluster size from 27 to 8 μm, but they were unable to achieve a homogeneous dispersion of MWCNT nanoparticles [62]. Therefore, the fraction of MWCNTs in the polymer matrix needs to be carefully controlled because of a trade-off between the mechanical performance and the functional properties of the resulting nanocomposites.

**Figure 7:**
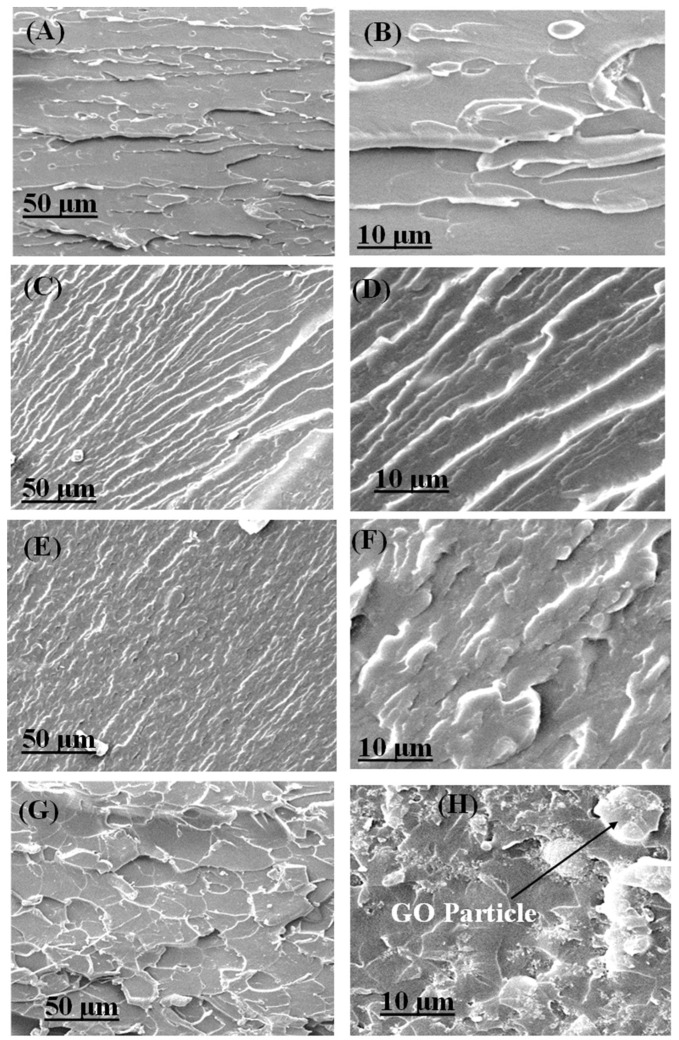
SEM micrographs of a fracture surface after tensile testing of (**A**,**B**) EP/GNP 0.2, (**C**,**D**) EP/GNP 0.4, (**E**,**F**) EP/GNP 0.6, and (**G**,**H**) EP/GO.

Figure 7 shows the fracture surfaces of EP/GNP and EP/GO nanocomposites. Incorporating graphene remarkably increased the roughness of surfaces. In addition, there are not any clear aggregations of nanoparticles. Figure 7 generally indicates that plastic deformation, crack deflection, and crack pinning were the prominent toughening mechanisms of the fracture surfaces. Once a crack encountered a rigid particle, the crack could be deflected by twisting or tilting, which required further energy and considerably improved the tensile features of the samples. The crack-pinning mechanism implies that as the crack propagated through the polymer matrix and faced nanoparticles, it probably arched around the nanoparticles and remained pinned at them [24,66]. Figure 7 shows that increasing the GNP incorporation induced crack deflection and considerably increased pinning. 

Figure 7A,B represents the fractured surfaces of the EP/GNP 0.2 wt% nanocomposite specimen, including crack deflection and crack pinning, which enhanced the nanocomposite’s fracture toughness and mechanical properties. Figure 7C,D indicates that, at EP/GNP 0.4 wt%, the roughness of the fracture surface had remarkably increased. The highest weight percent of GNP content (0.6 wt%) produced a rough fracture surface, with many microcracks that were forced to change direction to prevent crack propagation(Figure 7E,F). They are responsible for high energy dissipation and nanocomposite toughness [72,73].

Specimen EP/GO presents a quite different fracture morphology (Figure 7G,H), exhibiting many fracture ditches and obvious plastic deformations that led to a rougher fracture surface. This explains why the GO increased the roughness of the epoxy matrix by effectively preventing crack propagation [66]. The induced microcracks would not progress unless an increased force was applied. Thus, their paths had to frequently change direction and continue propagating toward the weak area in the nanocomposite. This phenomenon can be accompanied by the creation of extra-fractured surfaces, dissipating further energy [24,74]. Figure 7H shows a GO sheet (black arrow) tightly embedded in epoxy, revealing good interfacial interaction between the graphene oxide sheets and the epoxy matrix.

**Figure 8:**
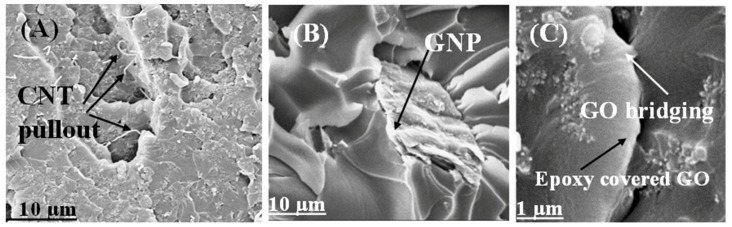
SEM micrographs of (**A**) EP/CNT 0.4, (**B**) EP/GNP 0.4, and (**C**) EP/GO 0.4.

A thorough analysis of the fracture surface of the EP/CNT, EP/GNP, and EP/GO nanocomposites revealed interesting differences in the interfacial interaction among the embedded nanoparticles and the epoxy resin in these three systems (Figure 8). The MWCNT nanoparticles protruded cleanly from the fracture surface, indicating a weak interfacial bond (Figure 8A), while the GO sheets were thickly coated with adsorbed epoxy resin, indicating strong polymer matrix GO interactions (Figure 8C). There were no obvious pull-out GNP particles on the fracture surface of EP/GNP, but a weak interface between them and the polymer matrix was observed (Figure 8B). According to these findings, two main differences between GNP and oxidized GNP (GO) can be suggested. First, distortions induced by the oxygen functionalization and the extremely small thickness of the resulting GO sheets probably led to a wrinkled topology at the nanoscale. This nanoscale surface roughness can lead to improved mechanical interlocking with the polymer chains, resulting in better adhesion [32,75]. Second, there were some pendant hydroxyl groups on the GO surfaces that could form hydrogen or even covalent bands with the carbonyl groups of the epoxy resin [75]. Together with the high surface area and nanoscale surface roughness of GO, this surface chemistry led to stronger interfacial interactions with the epoxy resin, which significantly affected the characteristics of the polymer matrix [75]. We refer interested readers to our previous publication for the detailed characterization and discussion of the epoxy-GO possible reactions [68].

### 3.5. Secondary Mechanisms to Improve the Mechanical Characteristics of the Nanocomposites

Crack deflection and pinning are the possible primary reinforcing mechanisms for nanofillers, along with thermoplastic deformation. We observed the bridging effect, which is the secondary mechanism enhancing the mechanical characteristics of the nanocomposites (Figure 8C) [76,77]. The bridging of nanoparticles across the cracked matrix prevented further crack propagation. Figure 8C shows SEM micrographs of GO bridging for EP/GO 0.4 wt%. Strong adhesion between the nanofillers and polymer matrix implies the mechanical properties of the nanocomposites. Figure 8A,B show neat MWCNTs and GNPs without residual epoxy resin on them, indicating relatively poor adhesion at the interface of the epoxy resin and nanofillers. As Figure 8C shows, the GO sheet was completely covered with a matrix; therefore, we did not observe pull-out GO particles. That indicates improved adhesion of the epoxy matrix/GO sheet interface. During the curing process, the number and size of graphene agglomerates and MWCNT clusters often increased because of (i) the Van der Waals forces between nanoparticles and (ii) phase separation between nanofillers and polymer matrix. The induced agglomerations and clusters yielded insufficient interface area and poor adhesion between the carbon-based nanomaterials and polymers, leading to a weakening of the reinforcing or toughening effect of MWCNTs and GNPs. In our study, the TEM and SEM images revealed that dispersing nanoparticles in the hardener produced a self-driving force to separate and exfoliate the nanoparticles.

### 3.6. Thermomechanical Properties

**Figure 9:**
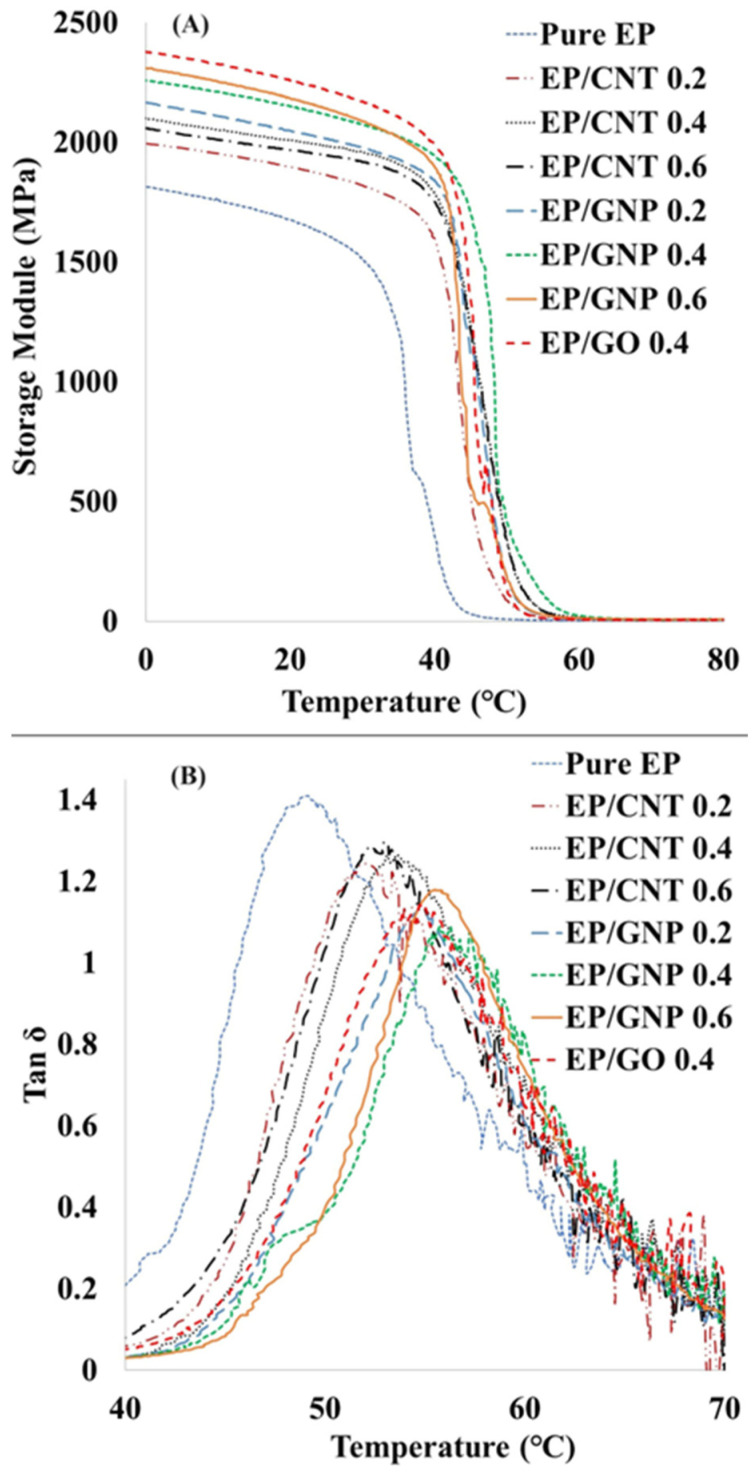
Dynamic mechanical properties of pure EP and nanocomposites: (**A**) storage modulus and (**B**) the loss factor of samples.

Dynamic mechanical analysis (DMA) was used to study the thermomechanical properties of the composites. The results are provided in Figure 9 and Table 4. The results show that the storage modulus of the pure epoxy in the glassy state at 0 °C was about 1810 MPa. This figure increased significantly for the nanocomposites. Table 4 shows that incorporating 0.2 wt% and 0.4 wt% of MWCNT increased the epoxy storage modulus to about 1990 and 2100 MPa, respectively, while loading 0.6 wt% of MWCNT decreased the storage modulus to 2055 MPa. That can be explained by the formation of MWCNT clusters, as already observed under SEM analysis.

Unlike with the MWCNT nanocomposites, the storage modulus continuously increased by increasing the GNP content. Increasing the GNP loading gradually increased the storage modulus of the nanocomposites to 2160, 2247, and 2300 MPa for EP/GNP 0.2, EP/GNP 0.4, and EP/GNP 0.6, respectively. In the glassy region, the storage modulus yielded an increase of about 28% for EP/GNP 0.6, whereas the incorporation of GO sheets in the epoxy increased the storage modulus to around 2353 MPa. 

Table 4 shows that by incorporating 0.4 wt% of nanoparticles in samples EP/CNT 0.4, EP/GNP 0.4, and EP/GO 0.4, the storage module of epoxy resin increased by around 17, 25, and 31%, respectively. This considerable effect of GO nanosheets on storage modulus, compared to GNP, can probably be attributed to the compatibility between the epoxy and GO sheets due to the oxygen functional groups on the GO nanoparticles, leading to high exfoliation, well dispersion of GO nanosheets, and strong epoxy GO interface interactions [56,78]. Some authors have shown that incorporating GO nanoparticles in epoxy a negligible effect on the epoxy’s storage modulus because of the aggregation of nanoparticles in the epoxy resin [79,80]. In contrast, we achieved a considerable increase in the storage modulus of epoxy nanocomposites.

**Table 4:** Storage modulus and glass transition temperature (Tg) values of pure epoxy and nanocomposites.

Sample	Pure EP	EP/CNT 0.2	EP/CNT 0.4	EP/CNT 0.6	EP/GNP 0.2	EP/GNP 0.4	EP/GNP 0.6	EP/GO 0.4
Storage modulus at 0 °C (MPa)	1810	1990	2100	2055	2160	2247	2300	2353
Tg (°C)	49	52	53	54	54	55	56	54

Table 4 gives the Tg values determined from the peak of the tan delta curves (Figure 9B). It shows that Tg gradually increased by increasing the content of nanoparticles in both EP/CNT and EP/GNP. This can be related to a rise in the mechanical interlocking between nanoparticles and epoxy resin. This phenomenon probably happened due to the wrinkled and folded structure of MWCNTs and GNPs, considerably restricting the mobility of polymer chains [81,82]. Moreover, the Tg of EP/GNP 0.4 wt% (55 °C) was higher than the Tg of EP/GO 0.4 wt% (54 °C). This can be attributed to the chemical interaction or bonding between GO nanoparticles and the epoxy resin, which reduced the crosslinking density of the epoxy resin [36]. In our previous study, three main competing factors that can influence the Tg of the GO/epoxy nanocomposite were completely discussed [68].

### 3.7. Moisture Uptake

**Figure 10:**
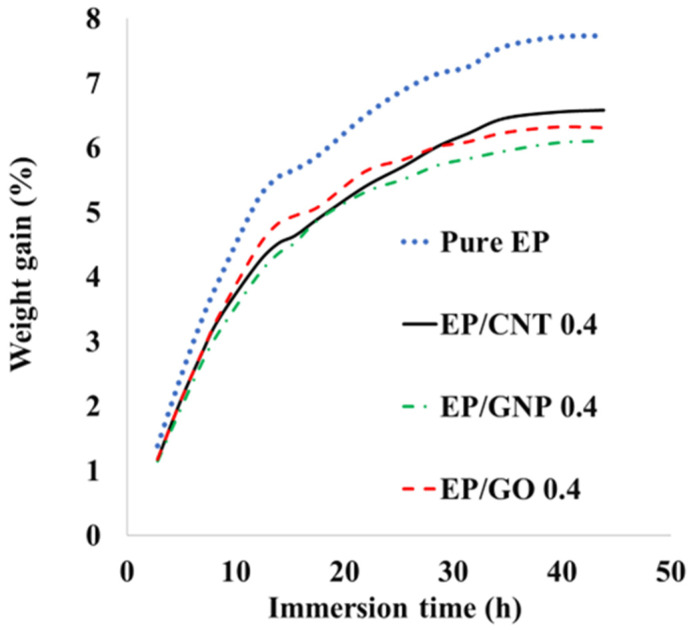
Moisture uptake vs. square root of time of pure epoxy and nanocomposite EP/CNT 0.4, EP/GNP 0.4, and EP/GO 0.4.

The moisture uptake of the pure epoxy resin and nanocomposites in EP/CNT 0.4, EP/GNP 0.4, and EP/GO 0.4 was measured by placing the specimens in a 50 °C water bath to accelerate the diffusion process. Figure 10 shows the absorption rate plotted as a function of the square root of time, demonstrating steady water absorption followed by a plateau, characteristic of Fickian behavior for pure resins and nanocomposites. The water uptake rate corresponds to the initial speed of diffusion inside the material. Figure 10 shows that incorporating carbon nanoparticles led to a slight decrease in the water uptake rate. In contrast, the incorporation of nanoparticles is expected to cause weak bonding at the interface between the nanomaterials and polymer matrix, allowing the water to diffuse at the interface. This phenomenon has been reported by some researchers who dispersed graphene nanoparticles in a polymer matrix. SEM micrographs have revealed micropores at the interface between the nanoparticles and polymer matrix [83,84]. According to Figure 10, adding just 0.4 wt% of GNP, GO, and MWCNT to the epoxy resin decreased the water absorption at saturation by about 26%, 22%, and 16%, respectively. These results reveal that graphene and GO, which are 2D nanosheets, function better as a barrier than MWCNTs, which are 1D nanoparticles. Interestingly, the moisture absorption of GO nanoparticles before reaching the saturation stage is higher than that of MWCNTs, and as it approaches the saturation state, it starts to decrease. It can also be seen that the moisture uptake is higher for GO nanocomposites compared to GNP nanocomposites, as the surface of GO sheets is more hydrophilic than the GNP surface [85]. The barrier performance of polymer matrix/GNP nanocomposites depends on GNP geometry and dispersion, as well as the interface in the polymer matrix [49].

## 4. Conclusions

In this research, a green (solvent-free and surfactant-free) method was applied to disperse MWCNTs, GNPs, and GO in epoxy resin. The various characterization techniques and experimental results reveal that the applied approach was effective in dispersing and improving the distribution uniformity of all three nanofillers. Nanocomposites with 0.2 wt% and 0.4 wt% of MWCNT exhibited relatively homogeneous dispersion, while a loading of 0.6 wt% of MWCNT resulted in the formation of particle clusters. In contrast to MWCNTs, GNPs displayed homogenous dispersion. Among the three nanofillers, GO displayed the most uniform dispersion in the prepared nanocomposites. The Young’s modulus, tensile strength, and toughness of the epoxy with a loading of just 0.4 wt% of GO were 2.5 GPa, 55.4 MPa, and 1.70 MJ m^−3^, respectively, corresponding to enhancements of 24%, 49%, and 160%, respectively. Analyzing the fracture surface of EP/CNT, EP/GNP, and EP/GO nanocomposites revealed an extraordinary difference in the interfacial interaction between the epoxy resin and nanofiller in these three systems. The storage module of the epoxy resin by incorporating 0.4 wt% of MWCNT improved by around 17%, while by adding 0.4 wt% GNP and 0.4 wt% GO, this figure increased by about 25 and 31%, respectively. Fractography analysis of fracture surfaces revealed the primary reinforcing mechanisms (crack deflection and penning) and the secondary mechanism (bridging effect) for enhancing the mechanical characteristics of the nanocomposites. The highest tensile strength and modulus storage were found in GO-reinforced epoxy nanocomposites, whereas GNP-reinforced epoxy nanocomposites represented the best durability properties. 

The findings of this research will help in improving the mechanical and viscoelastic properties, thermal stability, and long-term durability of the fiber-reinforced polymer (FRP) composite bars used as reinforcement for concrete structures instead of metal bars (steel), which are subject to electrochemical corrosion and accelerated degradation of reinforced concrete structures. Another interesting application for our research concerns the use of high-performance fiber-reinforced polymer (FRP) composite sheets or laminates for external reinforcement and the rehabilitation of existing concrete structures such as bridges, buildings, and maritime structures.

## Figures and Tables

**Table 1:** Nanoparticle specifications.

Nanoparticle	Multi-Wall CNT	Graphene Nanoplate
Morphology	Cylindrical	Planar
Purity (%)	>95	>99
Dimensions	Diameter: 50~100 nm Length: 5~20 μm	Diameter: 5~15 μm Thickness: 6~8 nm
Density (g/cm^3^)	1.9	2.1
Surface area (m^2^/g)	>70	80

**Table 2:** Composition of specimens.

Specimen	Nanofiller (wt%)	Epoxy/Hardener (g)
Pure epoxy (EP)	0	100
EP/CNT 0.2	0.2	100
EP/CNT 0.4	0.4	100
EP/CNT 0.6	0.6	100
EP/GNP 0.2	0.2	100
EP/GNP 0.4	0.4	100
EP/GNP 0.6	0.6	100
EP/GO 0.4	0.4	100

## Data Availability

All data, models, and code generated or used during the study appear in the submitted article.
